# Cefiderocol for the Treatment of Multidrug-Resistant Gram-Negative Bacteria: A Systematic Review of Currently Available Evidence

**DOI:** 10.3389/fphar.2022.896971

**Published:** 2022-04-12

**Authors:** Chuanhai Wang, Deqing Yang, Yifan Wang, Wentao Ni

**Affiliations:** ^1^ Department of Pulmonary and Critical Care Medicine, Shengli Oilfield Central Hospital, Dongying, China; ^2^ Department of Pharmacy, The Second Affiliated Hospital of Kunming Medical University, Kunming, China; ^3^ Department of Pulmonary and Critical Care Medicine, Peking University People’s Hospital, Beijing, China

**Keywords:** cefiderocol, multidrug resistant, carbapenem-resistant, gram-negative bacteria, systematic review

## Abstract

Cefiderocol is a novel synthetic siderophore-conjugated antibiotic that hijacks the bacterial iron transport systems facilitating drug entry into cells, achieving high periplasmic concentrations. This systematic review analyzed the currently available literature on cefiderocol. It summarized *in vitro* susceptibility data, *in vivo* antimicrobial activity, pharmacokinetics/pharmacodynamics (PK/PD), clinical efficacy, safety and resistance mechanisms of cefiderocol. Cefiderocol has potent *in vitro* and *in vivo* activity against multidrug-resistant (MDR) Gram-negative bacteria, including carbapenem-resistant isolates. But New Delhi Metallo-β-lactamase (NDM)- positive isolates showed significantly higher MICs than other carbapenemase-producing *Enterobacterales*, with a susceptible rate of 83.4% for cefiderocol. Cefiderocol is well-tolerated, and the PK/PD target values can be achieved using a standard dose regimen or adjusted doses according to renal function. Clinical trials demonstrated that cefiderocol was non-inferiority to the comparator drugs in treating complicated urinary tract infection and nosocomial pneumonia. Case reports and series showed that cefiderocol was a promising therapeutic agent in carbapenem-resistant infections. However, resistant isolates and reduced susceptibility during treatment to cefiderocol have already been reported. In conclusion, cefiderocol is a promising powerful weapon for treating MDR recalcitrant infections.

## Introduction

The spread of multi-drug resistant (MDR) bacteria is a great threat to public health. In 2017, the World Health Organisation (WHO) designated the ESKAPE pathogens (*Enterococcus faecium*, *Staphylococcus aureus*, *Klebsiella pneumoniae*, *Acinetobacter baumannii*, *Pseudomonas aeruginosa*, and *Enterobacter species*) as “priority status”, for which new antibiotics are urgently needed (De Oliveira et al., 2020; Tacconelli et al., 2017). Carbapenem-resistant gram-negative bacteria, including carbapenem-resistant *Enterobacteriaceae*, carbapenem-resistant *P. aeruginosa*, and carbapenem-resistant *A. baumannii*, are considered superbugs in healthcare settings. They are associated with resistance to nearly all classes of antibiotics commonly used in clinical settings. Due to the limited therapeutic options, polymyxins, a class of cationic peptide drugs abandoned in the last century due to high nephrotoxicity, are currently used to treat recalcitrant infections caused by carbapenem-resistant Gram-negative bacteria (Li et al., 2006). However, polymyxins are associated with unsatisfactory clinical outcomes and a high mortality rate among critically ill patients.

A few antibiotics being churned out of the drug discovery and development pipeline give hope of curbing antibiotic resistance. All bacteria, especially Gram-negative bacteria, need iron as an enzyme cofactor to catalyze redox reactions involved in various fundamental cellular processes. (Kramer et al., 2020). Taking advantage of this unique feature, cefiderocol, a novel synthetic siderophore-conjugated antibiotic has been developed, which can hijack the bacterial iron transport systems to facilitate the drug to enter cells, thereby achieving high periplasmic concentrations (Page, 2019). In addition, cefiderocol has a high affinity for penicillin binding proteins 3 (PBP3). The C-7 side chain in cefiderocol improves the transport across the bacterial outer membrane and can resist the hydrolysis by several β-lactamases (Aoki et al., 2018). Further, cefiderocol shows high *in vitro* potency against pathogenic carbapenem-resistant Gram-negative bacteria, with the minimum inhibitory concentration (MIC) lower than 4 mg/L for most *Enterobacterales*, *P. aeruginosa* and *A. baumannii* isolates (Yamano, 2019). It is approved by the Food and Drug Administration (FDA) to treat nosocomial pneumonia and complicated urinary tract infections (cUTIs).

Although cefiderocol is a promising antimicrobial agent against MDR Gram-negative bacteria, its efficacy in treating infections caused by carbapenem-resistant pathogens is uncertain (Simner and Patel, 2020). Furthermore, emergence of resistance has already been reported. Therefore, it is important to have a deep understanding of this novel siderophore-cephalosporin to promote rational use and thus reduce the emergence of resistance. This systematic review analyzes currently available literature evaluating the role of cefiderocol in treating MDR Gram-negative bacterial infections.

## Materials and Methods

### Search Strategy and Study Eligibility

This systematic review was performed in agreement with the Preferred Reporting Items for Systematic Reviews and Meta-Analyses (PRISMA) standards (Page et al., 2021). We systematically searched PUBMED, EMBASE and Cochrane Library databases from inception to 12 January 2022. The search terms included “cefiderocol”, “S-6492660” and “Fetroja”. Further, we reviewed the conference proceedings of the International Symposium on Antimicrobial Agents and Resistance (ISARR), Infectious Diseases Society of America (IDSA), and European Congress of Clinical Microbiology and Infectious Diseases (ECCMID) from the year 2015–2021 to reduce publication bias. Finally, we manually searched the reference lists of the included studies and systematic reviews to select relevant articles. This study was registered in the International Prospective Register of Systematic Reviews (Registration number: CRD42021286832).

Studies were considered eligible for inclusion if they reported on *in vitro* or *in vivo* antimicrobial activity, pharmacokinetics (PK) and pharmacodynamics (PD), clinical use and resistance of cefiderocol. Studies published in languages other than English or having duplicated data were excluded. The literature search and the study selection were carried out by two independent reviewers (WC and YD). Any disagreements were resolved by a third reviewer, and a final consensus was reached among all authors.

### Data Extraction and Quality Assessment

The following data were extracted by two independent reviewers: authors, publication year, details of the experimental methods or study design, number of tested strains, animals or patients, main characteristics of the tested strains or the study population, and the outcome measures. Cochrane risk of bias tool was used to assess the risk of bias of the included clinical trials.

### End-points

The primary end-point for *in vitro* studies on antimicrobial activity was the MICs and the susceptibility rate. The primary end-point in the animal studies was the *in vivo* efficacy. For the PK/PD studies, the primary end-point was the PK/PD targets. Finally, the primary end-points in clinical studies and trials were the clinical response and all-cause mortality.

### Quantitative Data Synthesis

Quantitative data were analyzed using Stata 14.0 (Stata Corporation, College Station, TX). Risk ratio (RR) and 95% confidence intervals (CI) were used as the eﬀect measures of outcomes for meta-analysis of clinical trials. Statistical heterogeneity among studies was assessed with the *I* ^*2*^ index (*I* ^*2*^ > 50% was considered substantial heterogeneity). The random-effect model was used when the heterogeneity was significant; in all other cases, the fixed eﬀect model was used.

## Results and Discussion

**FIGURE 1:**
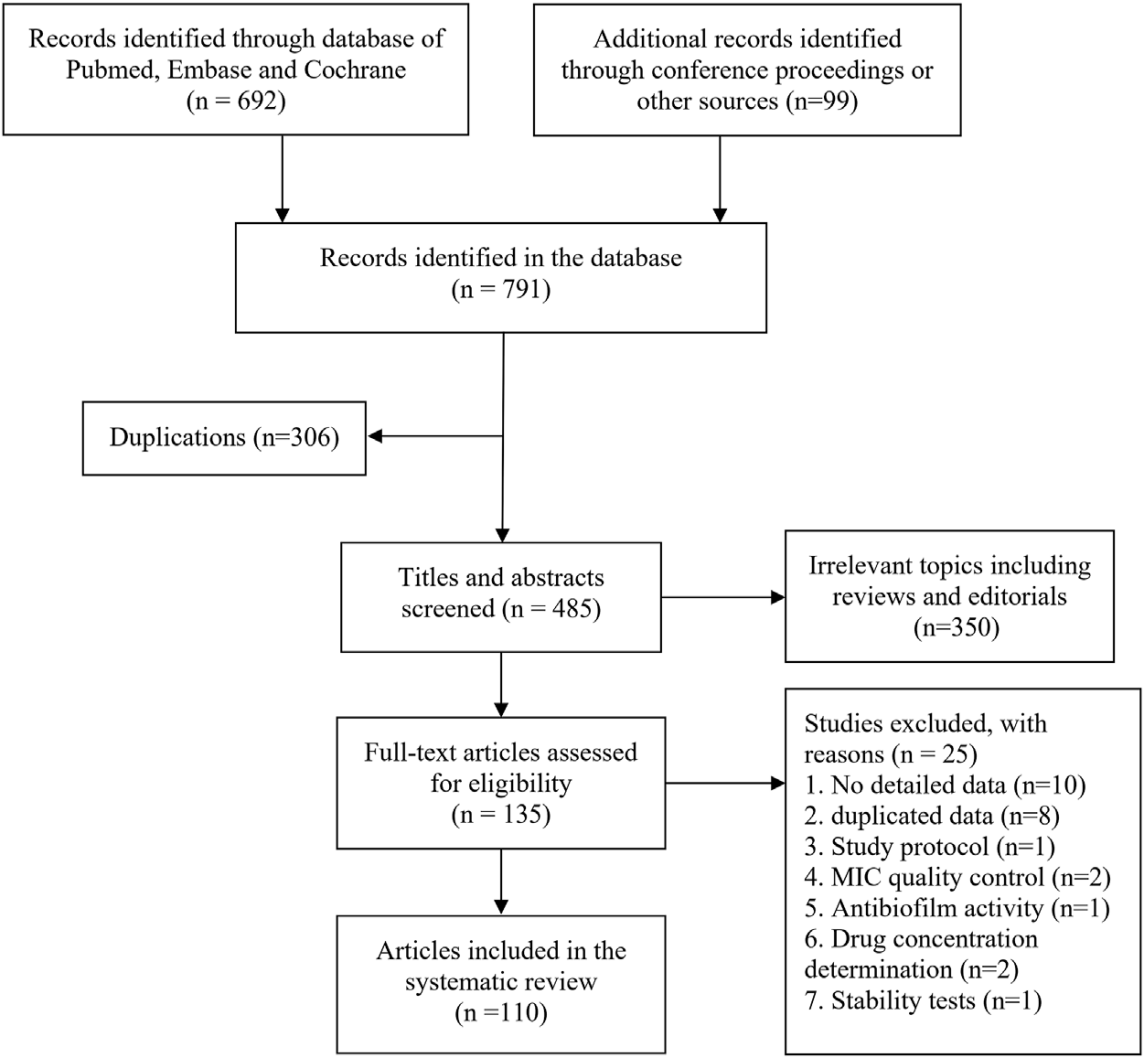
Flow diagram of literature search.

The literature search of databases yielded 692 citations. In addition, 99 conference proceedings on cefiderocol were included. Irrelevant studies were excluded after reviewing the full text. Finally, a total of 110 citations were included in this systematic review. Figure 1 shows a flow diagram of the literature search.

### *In vitro* Antimicrobial Activities

**FIGURE 2:**
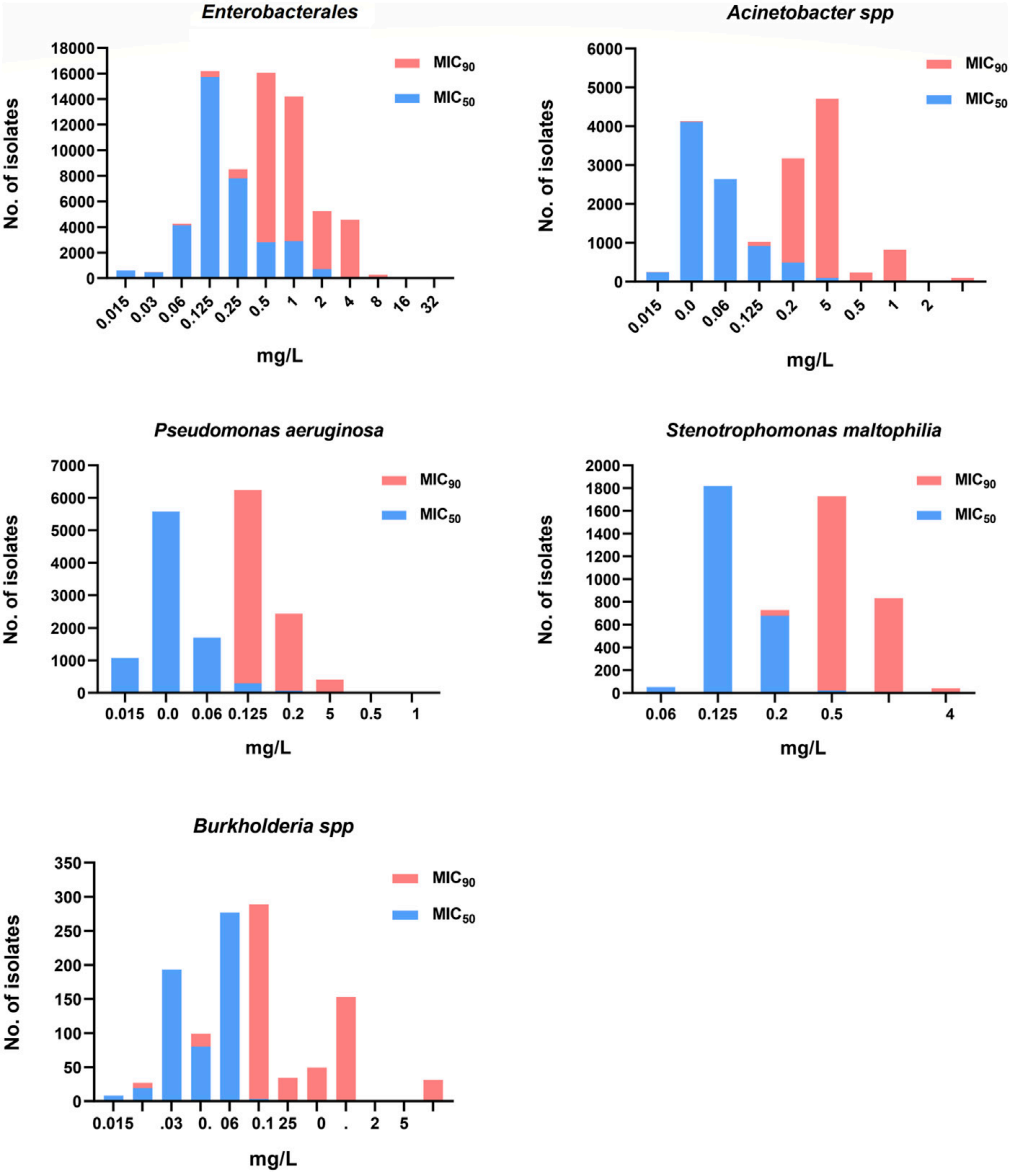
The cefiderocol MIC_50_ and MIC_90_ distribution of *Enterobacterales*, *Acinetobacter spp*, *Pseudomonas aeruginosa*, *Stenotrophomonas maltophilia* and *Burkholderia spp*.

Thirty-eight studies reporting on the *in vitro* activity of cefiderocol against Gram-negative bacteria were included (Ito et al., 2015; Ito et al., 2016; Kohira et al., 2015; Falagas et al., 2017; Dobias et al., 2017; Hackel et al., 2017; Kanazawa et al., 2017; Yamano et al., 2017; Hackel et al., 2018; Ito et al., 2018a; Jacobs et al., 2018; Karlowsky et al., 2019; Kazmierczak et al., 2019; Hsueh et al., 2019; Iregui et al., 2019; Albano et al., 2020; Biagi et al., 2020; Delgado-Valverde et al., 2020; Golden et al., 2020; Johnston et al., 2020; Kresken et al., 2020; Longshaw et al., 2020; Morris et al., 2020; Mushtaq et al., 2020; Rolston et al., 2020; Trebosc et al., 2020; Abdul-Mutakabbir et al., 2021; Bhagwat et al., 2021; Bianco et al., 2021; Burnard et al., 2021; Cercenado et al., 2021; Gant et al., 2021; Candel et al., 2022; Johnston et al., 2021; Lee et al., 2021; Liu et al., 2021; Stracquadanio et al., 2021; Zalacain et al., 2021). A total of 53,416 isolates, including 34,805 *Enterobacterales*, 8297 *P. aeruginosa*, 7249 *Acinetobacter spp*, 2508 *Stenotrophomonas maltophilia* and 549 *Burkholderia spp*, mainly collected from North America, Europe and East Asia excluding Chinese mainland, were tested for cefiderocol susceptibility. The distribution of MIC_50_ and MIC_90_ for the significant pathogens is shown in Figure 2. Most studies reported that the MIC_90_ of cefiderocol for *Enterobacterales* ranged between 0.5 and 4 mg/L. Two studies including 393 isolates reported a MIC_90_>4 mg/L for *Enterobacterales*, indicating that cefiderocol had a susceptibility rate lower than 90% (Albano et al., 2020; Morris et al., 2020). The MIC_90_ of cefiderocol in *Acinetobacter spp* was similar to that of *Enterobacterales*. However, six studies including 920 isolates, reported a MIC_90_ higher than 4 mg/L (Ito et al., 2015; Hackel et al., 2018; Hsueh et al., 2019; Albano et al., 2020; Morris et al., 2020; Trebosc et al., 2020). The MIC_90_ for *P. aeruginosa*, *S. maltophilia* and *Burkholderia spp* was lower than *Enterobacterales* and *Acinetobacter spp*, suggesting a higher sensitivity to cefiderocol.

**FIGURE 3:**
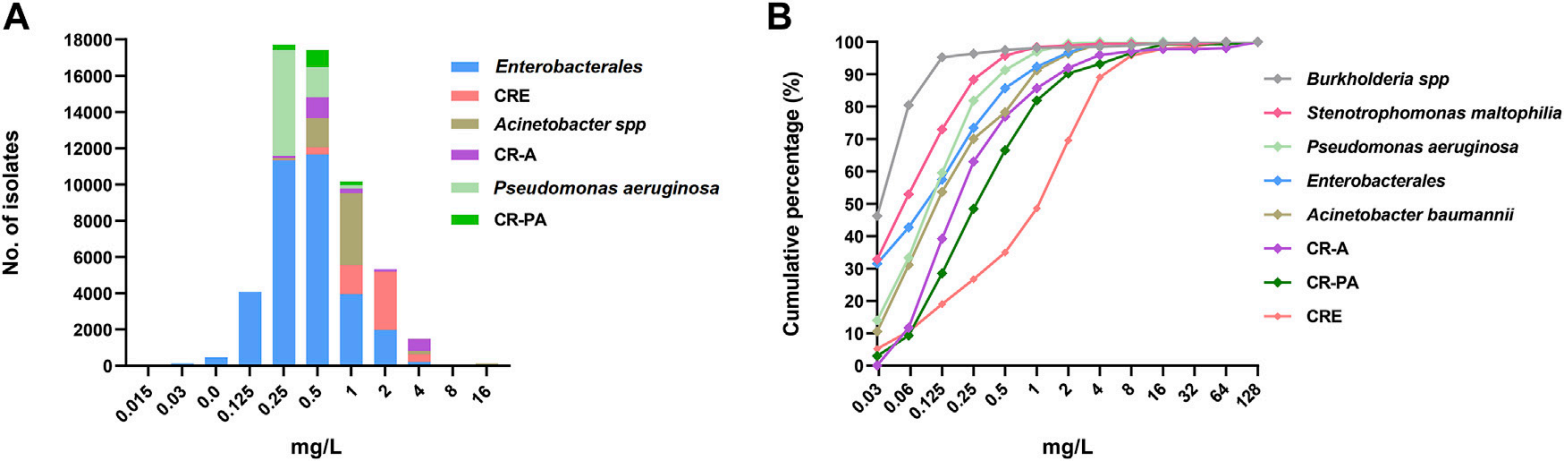
The cefiderocol MIC profile of *Enterobacterales*, *Acinetobacter spp* and *Pseudomonas aeruginosa* with or without carbapenem-resistance. **(A)**, the MIC_90_ distribution of the three Gram-negative bacteria; **(B)**, the cumulative curves of MICs of 9305 isolates (n = 13). CRE, carbapenem-resistant *Enterobacterales*; CR-A, carbapenem-resistant *Acinetobacter spp*; CR-PA, carbapenem-resistant *P. aeruginosa*.

Further, the MIC_90_ distribution for carbapenem-resistant isolates was compared with that of the “putative carbapenem-susceptible” isolates (data obtained from studies that did not report on susceptibility to carbapenems). As show in Figure 3A, the MIC_90_ for carbapenem-resistant isolates, especially the *Enterobacterales* and *Acinetobacter spp*, was higher than that for the ‘putative carbapenem-susceptible’ isolates. The MIC_90_ for carbapenem-resistant *Enterobacterales* (CRE) was higher than that of carbapenem-resistant *Acinetobacter spp* and carbapenem-resistant *P. aeruginosa*. The specific MIC values for 9305 isolates were obtained from 13 studies. The cumulative MIC distribution curves for cefiderocol also showed that the MICs for carbapenem-resistant isolates were higher than for the ‘putative carbapenem-susceptible’ isolates (Figure 3B).

**FIGURE 4:**
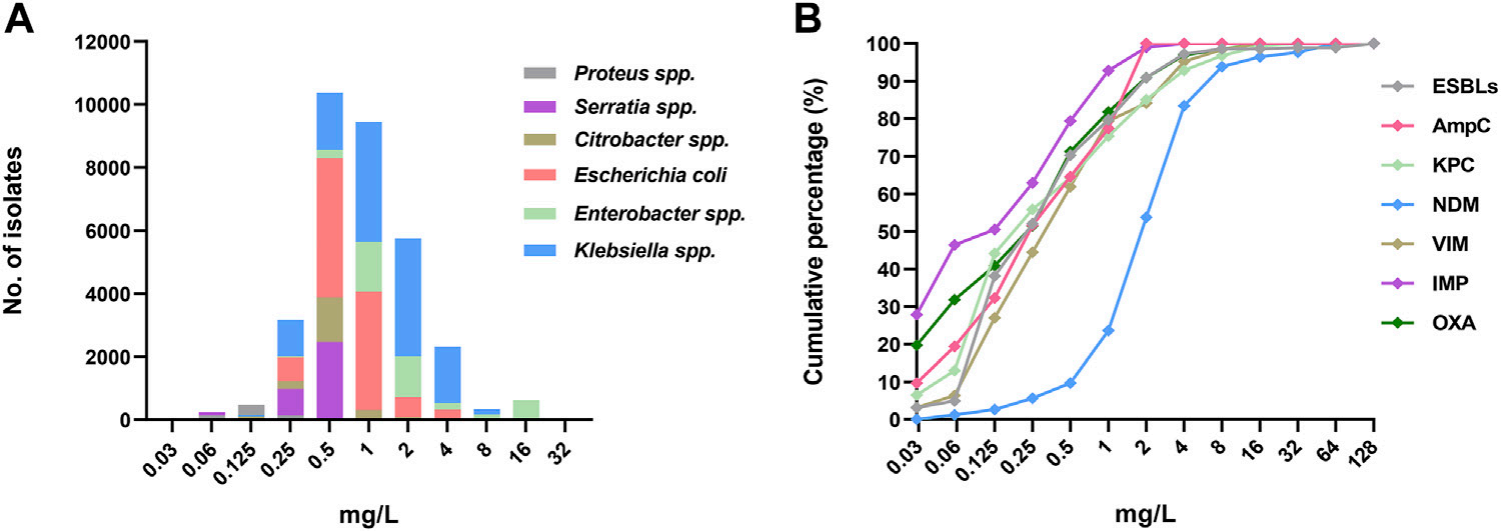
The cefiderocol MIC profile of different species of *Enterobacterales*, **(A)**, the MIC_90_ distribution; **(B)**, the cumulative curves of MICs of 1264 isolates (n = 15) harboring different β-lactamase.

The MIC_90_ distribution for different *Enterobacterales* species is shown in Figure 4A. The MIC_90_ for *Enterobacter spp* and *Klebsiella spp* were higher than for others species. Further, the distribution of MICs for *Enterobacterales* (1264 isolates) harboring different β-lactamase genes were obtained from 15 studies and analyzed. As shown in Figure 4B, the MICs for New Delhi metallo- β-lactamase (NDM) positive isolates were significantly higher than those harboring other β-lactamase genes, with a susceptibility rate of 83.4% for cefiderocol.

In addition, four studies investigate the synergistic *in vitro* activity of cefiderocol combined with other antimicrobial agents against Gram-negative bacteria (Tsuji et al., 2016; Yamano et al., 2020a; Biagi et al., 2020; Abdul-Mutakabbir et al., 2021). Abdul-Mutakabbir, et al. assessed the combination of β-lactamase inhibitors (BLIs) on reversing cefiderocol resistance for MDR *A. baumannii* (the MICs for cefiderocol were 16–32 mg/L) (Abdul-Mutakabbir et al., 2021). They found that the addition of BLIs resulted in lower MIC values. Avibactam exerted the strongest effects with 4–64 folds reduction in the MIC values (0.5–8 mg/L) (Abdul-Mutakabbir et al., 2021). Another study by Yamano et al. reported that cefiderocol combined with avibactam or sulbactam showed synergistic activity against cefiderocol-resistant PER-producing *A. baumannii* (Yamano et al., 2020a). Besides, the two studies reported synergistic activity in combination therapy of cefiderocol-meropenem, cefiderocol-amikacin, cefiderocol-tigecycline, cefiderocol-minocycline and cefiderocol-ampicillin-sulbactam, even though the isolates showed resistance to both cefiderocol and meropenem/amikacin (Yamano et al., 2020a; Abdul-Mutakabbir et al., 2021). Biagi, et al. used time-kill assays to show the synergy when cefiderocol combined with levofloxacin, minocycline, polymyxin B, or TMP-SMZ against S. maltophilia were 44.4% (4/9), 66.7% (6/9), 55.5% (5/9), and 66.7% (6/9), respectively (Biagi et al., 2020). Using the checkboard method, Tsuji, et al. showed that cefiderocol combined with meropenem, ciprofloxacin, and amikacin showed synergistic effects against *A. baumannii*, *P. aeruginosa* and *K. pneumoniae* (Tsuji et al., 2016).

The Clinical and Laboratory Standards Institute recommends the use of iron-depleted cation-adjusted Mueller–Hinton broth (ID-CAMHB) for the determination of cefiderocol MICs (Clinical and Laboratory S, 2020). Among the 40 *in vitro* studies, 6 did not report the concrete methodologies used for determination of the MICs of cefiderocol (Ito et al., 2015; Ito et al., 2018a; Rolston et al., 2020; Trebosc et al., 2020; Abdul-Mutakabbir et al., 2021; Bhagwat et al., 2021), and the others used iron-depleted broth medium in the MIC testing. There may be potential some bias in the pooled results of susceptibility tests.

### PK/PD and Animal Studies

Twenty-five studies investigated the characteristics of PK and/or PD of cefiderocol (Katsube et al., 2016; Katsube et al., 2017; Matsumoto et al., 2017; Monogue et al., 2017; Ghazi et al., 2018a; Ghazi et al., 2018b; Katsube et al., 2018; Kawaguchi et al., 2018; Saisho et al., 2018; Katsube et al., 2019a; Kidd et al., 2019a; Katsube et al., 2019b; Kidd et al., 2019b; Chen et al., 2019; Miyazaki et al., 2019; Nakamura et al., 2019; Stainton et al., 2019; Matsumoto et al., 2020; Ota et al., 2020; Gill et al., 2021; Katsube et al., 2021; Kawaguchi et al., 2021; Kobic et al., 2021; König et al., 2021; Nakamura et al., 2021). A phase I study including healthy Japanese and Caucasian volunteers showed exhibit linear PK at doses of up to 2,000 mg, with low to moderate interindividual variability (Saisho et al., 2018). Cefiderocol was mainly eliminated unchanged in urine (Miyazaki et al., 2019), with metabolism contributing to less than 10% elimination (Saisho et al., 2018). Since cefiderocol is primarily eliminated through the renal route, renal impairment alters area under the plasma concentration-time curve (AUC), total drug clearance from plasma (CL) and terminal half-life (t_1/2_), without significantly affecting the maximum plasma concentration (C_max_) (Katsube et al., 2017; Kawaguchi et al., 2018; Kobic et al., 2021; König et al., 2021). Kawaguchi, et al. evaluated the PK of cefiderocol in patients with pneumonia, bloodstream infection/sepsis, or complicated urinary tract infection, finding that no other factors, including infection sites and mechanical ventilation, were statistically significant covariates in the population PK analysis (Kawaguchi et al., 2021). The intrapulmonary PK of cefiderocol was further evaluated in healthy adult subjects (n = 20) and mechanically ventilated patients with pneumonia (n = 7) (Katsube et al., 2019a; Katsube et al., 2021). In the healthy subjects, the geometric mean concentrations of cefiderocol in epithelial lining fluid (ELF) were 13.8, 6.7, 2.8 and 1.4 mg/L at 1, 2, 4 and 6 h from infusion initiation, respectively. The ratios of ELF concentration to total plasma concentration over 6 h ranged from 0.093 to 0.12 (Katsube et al., 2019a). In the mechanically ventilated patients with pneumonia, the ELF concentration was 7.63 mg/L at the end of infusion and 10.40 mg/L at 2 h after the end of infusion. The ratios of ELF concentration to total plasma concentration ranged from 0.09 to 0.42 at the end of infusion and 0.44–0.82 at 2 h after the end of the infusion (Katsube et al., 2021). These results suggest that cefiderocol can penetrate into the ELF.

Kidd et al. established neutropenic murine thigh infection models with iron overload and deficiency (Kidd et al., 2019a). They showed that the plasma concentrations of cefiderocol were similar in the iron overload models and the control group (Kidd et al., 2019a). However, the plasma concentrations in the iron-depleted mice were lower than that in the control group, indicating that *in vivo* iron deficiency might alter the PK of cefiderocol (Kidd et al., 2019a). Moreover, Katsube, et al. showed that administration of cefiderocol did not significantly affect OAT1, OAT3, OCT1, OCT2, and MATE2-K drug transporters, suggesting no clinically significant drug-drug interaction potential via the transporters (Katsube et al., 2018).

Animal studies demonstrated that cefiderocol exhibited time-dependent PD similar to other β-lactam antibiotics (Ghazi et al., 2018a; Nakamura et al., 2019). Considering that the bactericidal activity of β-lactam antibiotics can be enhanced by prolonging the infusion time, the recommended standard dose regimen for cefiderocol is 2g q8h with a 3-h infusion (Fetroja (Cefiderocol), 2021). An *in vitro* PK/PD study showed that the standard dose could completely kill meropenem-resistant gram-negative isolates showing cefiderocol MICs of 0.5–4 g/ml within 24 h (Matsumoto et al., 2020). Nine animal studies using neutropenic murine thigh models or respiratory tract infection models mimicking humanized exposures (2g q8h with a 3-h infusion) showed a >1 log_10_ reduction in bacterial colony forming units (CFU) of most Gram-negative bacteria with MICs ≤4 g/ml, but not for the isolates with MICs ≥ 8 mg/L (Supplementary Table S1) (Matsumoto et al., 2017; Monogue et al., 2017; Ghazi et al., 2018b; Kidd et al., 2019b; Chen et al., 2019; Stainton et al., 2019; Ota et al., 2020; Gill et al., 2021; Nakamura et al., 2021).

Monte-Carlo simulations based on population PK models in accounting for protein binding of 57.8% showed the standard dose yielded >90% probability of target attainment (PTA) for 75% T_*f>MIC*_ for an MIC ≤4 g/ml for adults or pediatric patients with normal renal function (Katsube et al., 2016; Katsube et al., 2019b). The dose of cefiderocol should be adjusted according to the renal function and whether patients are on hemodialysis or continuous renal replacement therapy. Another Monte-Carlo simulation study found >90% PTA for 100% T_*f>MIC*_ for an MIC ≤4 g/ml in different infections and renal function groups could be achieved, except for bloodstream infection/sepsis patients with normal renal function (85%) (Kawaguchi et al., 2021).

### Clinical Trials

By far, the clinical efficacy of cefiderocol has been investigated in three randomized controlled trials (RCTs), including one phase II trial (APEKS-cUTI) and two phase III trials (APEKS-NP and CREDIBLE-CR) (Portsmouth et al., 2018; Bassetti et al., 2021; Wunderink et al., 2021). The baseline demographics and pathogen distribution of the study populations are shown in Supplementary Table S2. Further, the risk of bias of the three RCTs is shown in Supplementary Figure S1.

The APEKS-cUTI trial compared the efficacy of cefiderocol versus imipenem/cilastatin in the treatment of complicated urinary tract infections (cUTIs) (Portsmouth et al., 2018). The primary endpoint included both clinical and microbiological outcomes at test of cure (7 days after treatment cessation). A total of 371 patients [cefiderocol (n = 252); imipenem/cilastatin (n = 119)] with qualifying Gram-negative uropathogen (≥1 × 10⁵ CFU/mL) were included in the primary efficacy analysis. The most common pathogens in both groups were *Escherichia coli* and *K. pneumoniae*. The primary efficacy endpoint was achieved by 72.6% (183/252) patients in the cefiderocol group and 54.6% (65/119) patients in the control group with an adjusted treatment difference of 18.6% (95% CI: 8.2–28.9, *p* = 0.0004). These results suggested that cefiderocol was non-inferior to imipenem/cilastatin for cUTIs.

The APEKS-NP trial evaluated the efficacy of cefiderocol versus meropenem with high-dose, extended-infusion (2g q8h with a 3-h infusion) for nosocomial pneumonia (hospital-acquired pneumonia, ventilator-associated pneumonia, or health-care-associated pneumonia) caused by gram-negative bacteria (Wunderink et al., 2021). A total of 292 patients were included in the modified intention-to-treat population, with 145 in the cefiderocol group and 147 in the meropenem group. The most common pathogens were *K. pneumoniae* followed by *P. aeruginosa* and *A. baumannii*. There were no significant differences in the primary endpoint (all-cause mortality at day 14) observed between two groups (12.4% in cefiderocol versus 11.6% in the meropenem group, the adjusted difference was 0.8%, 95% CI: 6.6–8.2%).

The CREDIBLE-CR trial evaluated the efficacy of cefiderocol versus the best available therapies (mainly colistin-based regimens) in adults with severe infections caused by carbapenem-resistant Gram-negative bacteria. This study enrolled 150 patients with nosocomial pneumonia (n = 67, 44.6%), bloodstream infection/sepsis (n = 47, 31.3%) or cUTIs (n = 36, 24.0%) (Bassetti et al., 2021). The most common pathogens were carbapenem-resistant *Acinetobacter spp* (n = 56), *K. pneumoniae* (n = 39) and *P. aeruginosa* (n = 22), with cefiderocol MIC_90_ of 1 g/ml, 4 mg/ml, and 2 mg/ml, respectively. The clinical cure rate for nosocomial pneumonia or bloodstream infection/sepsis and the microbiological eradication rate in cUTIs were not significantly different between the two groups. However, the mortality rate in the cefiderocol group [33.7% (34/101)] was higher than that of the control group [18.3% (9/49)]. Most deaths due to treatment failure in the cefiderocol group occurred in patients with infection due to *Acinetobacter spp* (13/16). Only one death (1/4) due to *Acinetobacter spp* infections was reported in the control group. In patients with infections due to other bacteria, no differences in mortality rates were noticed between the two groups. The efficacy of cefiderocol for treating MDR *Acinetobacter spp* infections deserves further clinical investigation.

A recent meta-analysis pooled the results of the three studies, and found no significant difference between cefiderocol and the comparators in terms of clinical response, microbiological response, all-cause mortality and adverse events (Hsueh et al., 2021). The most common reported adverse events were nausea, diarrhea, rash, elevated aminotransferase levels, and hypokalemia. Besides, a phase I study conducted in healthy persons showed that therapeutic doses of cefiderocol had no apparent effect on the QT interval.

**FIGURE 5:**
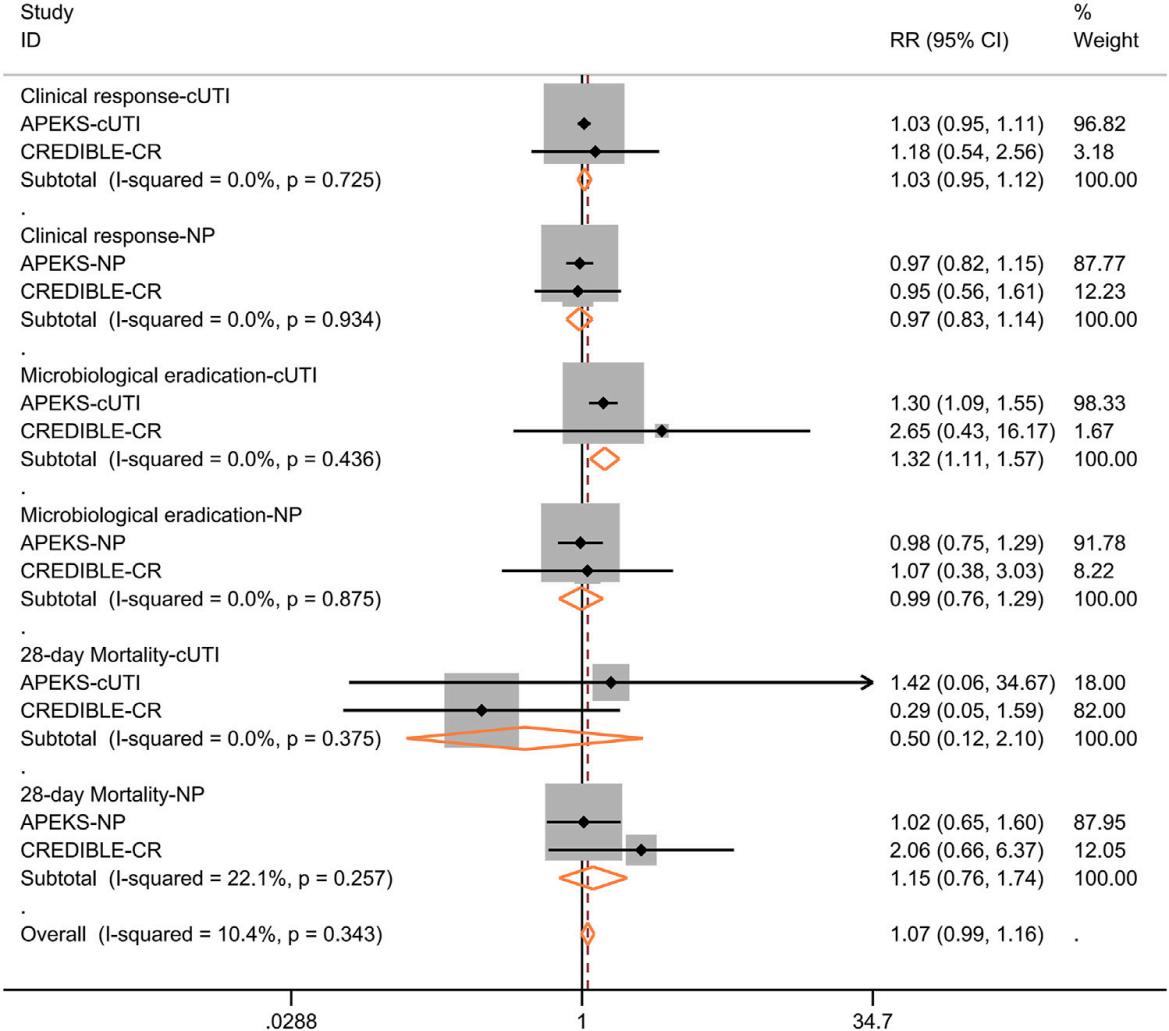
Forest plot for the pooled analysis of clinical response at test of cure, microbiological response and 28-days all-cause mortality between cefiderocol and comparators for the treatment of complicated urinary tract infection (cUTI) or nosocomial pneumonia (NP).

**FIGURE 6:**
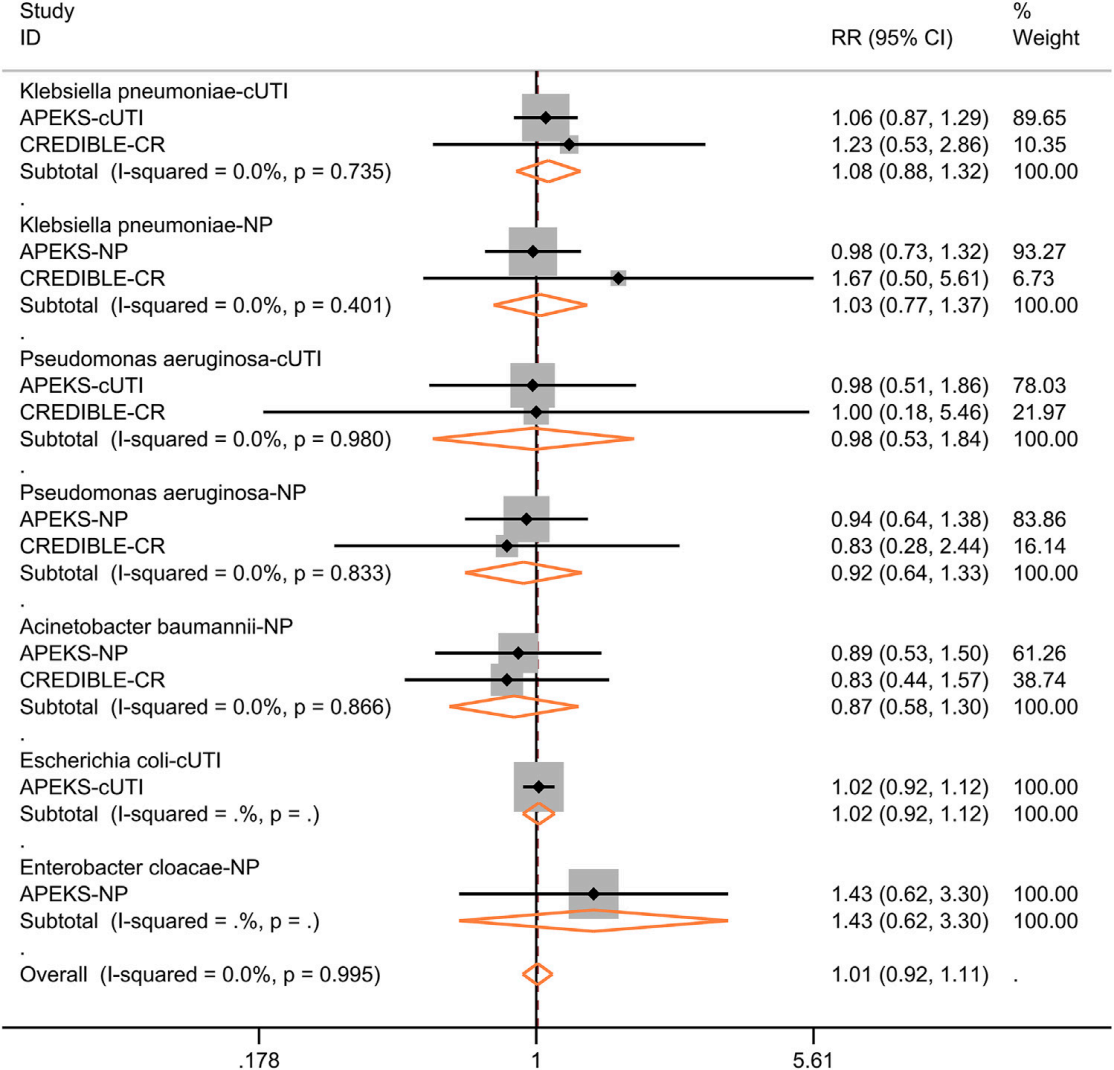
Forest plot for the pooled analysis of clinical response at test of cure between cefiderocol and comparators for the treatment of infections caused by specific pathogens. Complicated urinary tract infection, cUTI; Nosocomial pneumonia, NP.

**FIGURE 7:**
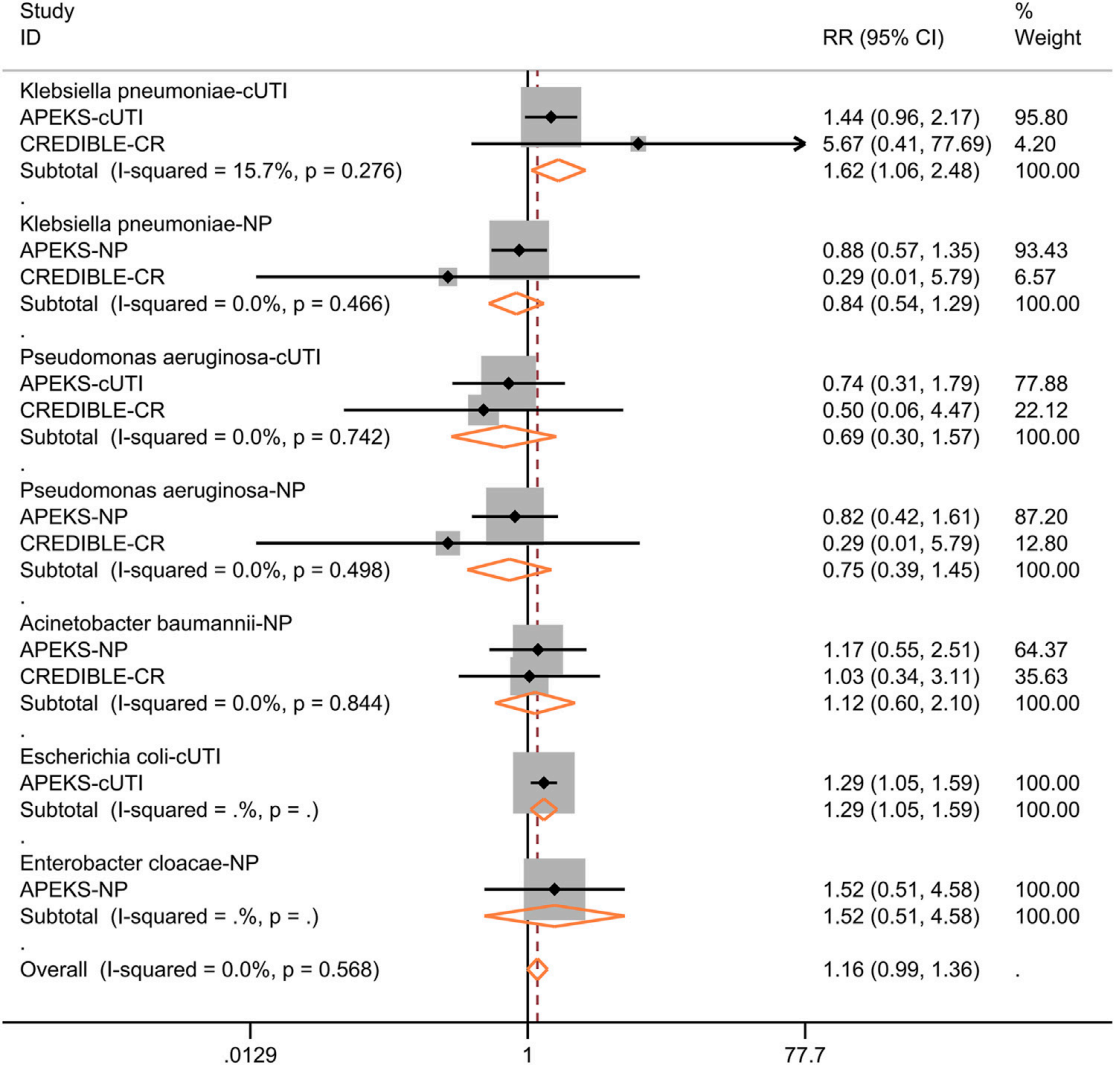
Forest plot for the pooled analysis of microbiological eradication at test of cure between cefiderocol and comparators for the treatment of infections caused by specific pathogens. Complicated urinary tract infection, cUTI; Nosocomial pneumonia, NP.

We further performed subgroup analysis for the efficacy of cefiderocol in treating nosocomial pneumonia or cUTI. As shown in Figure 5, the clinical response at the time of test of cure, microbiological response, 28-days all-cause mortality were not significantly different between cefiderocol and comparators. The subgroup analysis for different pathogens showed the clinical response was similar in the two groups (Figure 6). In the subgroup analysis for microbiological eradication of different pathogens (Figure 7), the cefiderocol group had higher microbiological eradication when treating cUTI caused by *K. pneumoniae* (RR = 1.6, 95% CI: 1.1–2.5, *I* ^*2*^ = 15.7%) or *E. coli* (RR = 1.3, 95% CI: 1.1–1.6).

### Case Reports and Case Series

We identified 30 case reports and case series including 78 patients who had recalcitrant infections caused by MDR Gram-negative bacteria, and treated with salvage treatment or compassionate use of cefiderocol in real-world settings (Edgeworth et al., 2019; Stevens and Clancy, 2019; Trecarichi et al., 2019; Alamarat et al., 2020; Contreras et al., 2020; Dagher et al., 2020; Kufel et al., 2020; Lampejo et al., 2020; Oliva et al., 2020; Siméon et al., 2020; Zingg et al., 2020; Bavaro et al., 2021a; Bavaro et al., 2021b; Bleibtreu et al., 2021; Bodro et al., 2021; Borghesi et al., 2021; Carney et al., 2021; Chavda et al., 2021; Cipko et al., 2021; Falcone et al., 2021; Fratoni et al., 2021; Grande Perez et al., 2021; Grasa et al., 2021; Klein et al., 2021; König et al., 2021; Mabayoje et al., 2021; Martinez et al., 2021; Mc Gann et al., 2021; Warner et al., 2021; Zaidan et al., 2021). The detailed characteristics of these cases are summarized in Supplementary Table S3. Most patients were adult (74/78), and the most common reason for hospitalization were COVID-19, trauma and bone fracture, organ transplantation and cystic fibrosis, et al. The patients mostly had bloodstream infections (n = 26), lower respiratory tract infections (n = 24), including ventilator-associated pneumonia (n = 8), wound infections (n = 6), osteomyelitis (n = 5), and intra-abdominal infections (n = 4), caused mostly by *A. baumannii* (n = 33), *P. aeruginosa* (n = 25), *K. pneumoniae* (n = 12) and *Achromobacter spp* (n = 10). Eleven patients had polymicrobial infections.

Twenty-three studies including 47 patients, reported on therapy regimens given before using cefiderocol. Among them, 42 patients received colistin (polymyxin E) or polymyxin B-based therapies. Four patients received colistin monotherapy, and the other patients received polymyxin combination therapies. Tigeycline, meropenem and fosfomycin were the most common antibiotics used in combination therapy. The most frequent reasons for switching to cefiderocol based regimen was treatment failure (n = 36), and/or polymyxin-associated toxicity (n = 13) ([enal toxicity (n = 7), neurotoxicity (n = 4)]. Among the 73 patients with detailed cefiderocol-based regimens, 30 received cefiderocol monotherapy, and the others received combination therapy (mainly combined with polymyxins, tigecycline, fosfomycin, meropenem or ceftazidime-avibatam). The total clinical response, microbiological eradication and mortality rates were 73.1% (57/78), 74.3% (57/77), 24.4% (19/78), respectively. Cefiderocol associated adverse events were reported in six patients, including leukopenia (n = 2), thrombocytopenia (n = 2), acute kidney injury (n = 2). The clinical response of cefiderocol for treating cefiderocol-susceptible *A. baumannii*, *Enterobacterales* and *P. aeruginosa* were 85.2% (23/27), 100% (8/8) and 81.3% (13/16), respectively. These data supported the role of cefiderocol in treating MDR Gram-negative bacteria infections. Nevertheless, it should be noted that the pooled analysis results of case reports and case series were better than those of the CREDIBLE-CR trial, due to possible selection bias and/or publication bias.

### Resistant Mechanisms

Overall, the worldwide resistant rate (MIC > 8 mg/L) of MDR gram-negative bacteria for cefiderocol is quite low. However, clinical resistance has been reported. In the APEKS-NP and CREDIBLE-CR studies, a ≥4-fold MIC increase during the treatment was found in 4.4% (7/159) and 11.3% (12/106) isolates, respectively (Bassetti et al., 2021; Wunderink et al., 2021). Klein, et al. reported the development of high resistance within 21 days of cefiderocol therapy in a patient with intra-abdominal and bloodstream infections caused by carbapenemase-producing *Enterobacter cloacae* (Klein et al., 2021). In addition, Choby, et al. reported widespread cefiderocol heteroresistance in carbapenem-resistant *A. baumannii* (59%), *Klebsiella spp* (30%), and *S. maltophilia* (48%) (Choby et al., 2021). Though *in vitro* heteroresistance of bacteria has not been clinically validated to be predictive of clinical or microbiological outcomes *in vivo*, the presence of resistant subpopulation in heteroresistant isolates may be selected and predominates, ultimately resulting in cefiderocol resistance.

Various mechanisms are associated with reduced susceptibility to cefiderocol. Firstly, several studies showed that cefiderocol-resistant isolates often harbored genes encoding NDM, PER and VEB β-lactamases, suggesting that these β-lactamases may contribute to cefiderocol resistance (Ito et al., 2019; Yamano et al., 2020b; Kohira et al., 2020; Poirel et al., 2021). The addition of avibactam could significantly decrease the MICs of non-susceptible *A. baumannii* isolates (Abdul-Mutakabbir et al., 2021), suggesting the involvement of β-lactamases in resistance. Secondly, structural changes in AmpC and KPC β-lactamases could confer reduced susceptibility to the cefiderocol, ceftazidime-avibactam and other cephalosporins (Shields et al., 2020; Hobson et al., 2021; Simner et al., 2021). Thirdly, reduced expression or mutation of genes involving iron transport pathways, especially the siderophore receptor genes (*pirA*, *cirA*, et al.) are associated with cefiderocol resistance (Ito et al., 2018b; Yamano et al., 2020b; Yamano et al., 2020c; Malik et al., 2020; Klein et al., 2021; Streling et al., 2021). Lastly, two studies found that mutations in the target gene PBP-3 might contribute to cefiderocol resistance (Malik et al., 2020; Takemura et al., 2020).

## Conclusion

Cefiderocol shows extensive *in vitro* and *in vivo* activities against MDR Gram-negative bacteria, including carbapenem-resistant isolates. It is well tolerated and the PK/PD target can be achieved in most patients by using standard dosage (2g q8h) or adjusting doses according to the renal function. Clinical trials and case reports/series show that cefiderocol is a promising therapeutic option for carbapenem-resistant recalcitrant infections. Since resistant isolates have already been reported, cefiderocol should be used judiciously to prevent widespread resistance. More clinical data is still needed to testify its efficacy.
