# Erratum: Cefiderocol for the Treatment of Multidrug-Resistant Gram-Negative Bacteria: A Systematic Review of Currently Available Evidence

**DOI:** 10.3389/fphar.2022.976792

**Published:** 2022-08-18

**Authors:** Frontiers Production Office

**Affiliations:** Frontiers Media SA, Lausanne, Switzerland

**Keywords:** cefiderocol, multidrug resistant, carbapenem-resistant, gram-negative bacteria, systematic review

Due to a production error, the Funding statement was not provided. The correct Funding statement is as follows: This work was supported by the National Natural Science Foundation of China (81903672), Peking University People’s Hospital Research and Development Funds (RS2020-04), and China International Medical Foundation (Z-2018-35-2003). The funders had no role in study design, data collection and analysis, decision to publish, or preparation of the manuscript.

The publisher apologizes for this mistake. The original version of this article has been updated. Your article has been copyedited to ensure that we publish the highest quality work possible. Please check it carefully to make sure that it is correct and that the meaning was not lost during the process.

