# Supplementary material for: Cefiderocol for the Treatment of Multidrug-Resistant Gram-Negative Bacteria: A Systematic Review of Currently Available Evidence
Source: Front Pharmacol. 2022 Apr 12;13:896971. doi: 10.3389/fphar.2022.896971 (PMC9039133; doi:10.3389/fphar.2022.896971)
Supplement: Supplementary file 1 [file DataSheet1.PDF]

Table S1. In vivo efficacy of cefiderocol against Gram-negative bacteria with different susceptibility using various animal infection models

Reference	Published year	Pathogen	Infection model	Simulated dose	Test time after inoculation (h)	No. of isolates	Cefiderocol MIC (mg/L)	In vivo efficacy (compared with the initial inoculum)		
								> 2 log_{10} reduction	1-2 log_{10} reduction	< 1 log_{10} reduction
Matsumoto S, et al.[66]	2017	Cephalosporin-susceptible *P. aeruginosa*	Immunocompetent-rat respiratory tract infection models	2g Q8h	96	1	0.5	1	0	0
		MDR *P. aeruginosa*				1	2	1	0	0
		MDR *A. baumannii*				2	0.125-2	2	0	0
		Carbapenem-resistant *K. pneumoniae*				2	4-8	2	0	0
Monogue M, et al.[67]	2017	*K. pneumoniae*	Neutropenic murine thigh model	2g Q8h	24	7	0.5-2	2	2	3
						13	4	0	5	8
						6	8-64	0	0	6
		E.coli				10	0.5-2	3	3	4
						1	4	0	1	0
						2	16- > 64	0	0	2
		A. baumannii				15	0.12-2	4	6	5
						1	4	1	0	0
						19	8- > 256	0	0	19
		P. aeruginosa				16	0.25-2	4	6	6
						4	4	4	0	0
						1	8	0	0	1
Ghazi I, et al.[68]	2017	*P. aeruginosa*	Neutropenic murine thigh model	2g Q8h	24	8	0.06-0.5	7	1	0
Stainton S, et al.[69]	2018	*A. baumannii*	Neutropenic murine thigh model	2g Q8h	24	2	0.5-1	1	1	0
						1	4	0	0	0

						1	16	0	0	1
		P. aeruginosa				2	2-4	1	1	0
		E.coli				2	1	0	1	1
		K. pneumoniae				3	4	0	1	2
						1	8	0	0	1
Chen IH, et al. [70]	2019	Stenotrophomonas maltophilia	Neutropenic murine thigh model	2g Q8h	24	24	0.015-0.5	21	3	0
Kidd J, et al.[71]	2019	*Enterobacterales*	Neutropenic murine thigh model	2g Q8h	24	9	0.25-2	4	3	2
						5	4	0	4	1
						2	16	0	0	2
		A. baumannii				5	0.25-2	5	0	0
						1	4	1	0	0
						2	16-64	0	1	1
		P. aeruginosa				4	0.5-2	2	2	0
						2	4	2	0	0
						1	8	0	0	1
Nakamura R, et al.[72]	2020	*S. maltophilia*	Neutropenic murine lung infection model	30mg/kg	24	2	0.06-0.5	2	0	0
			Immunocompetent rat respiratory-infection model	2g Q8h	96			2	0	0
Ota A, et al.[73]	2020	*A. baumannii*	Neutropenic ventilator-associated pneumonia mouse model	2g Q8h	24	1	0.5	1		
		P. aeruginosa			24	1	0.008	1		
Gill C, et al.[74]	2021	*S. maltophilia*	Neutropenic murine thigh model	2g Q8h	24	1	8	0	0	1

Table S2. Baseline demographic characteristics of the study populations and distribution of pathogens in the three multicenter RCTs.

Study, year published	Infection population	No. of patients (ITT population)		Treatment		Pathogens											
						E. coli		*K. pneumoniae*		*Acinetobacter spp*		*P. aeruginosa*		*E. cloacae*		Others	
		CFDC	Com	CFDC	Com	CFDC	COM	CFDC	COM	CFDC	COM	CFDC	COM	CFDC	COM	CFDC	COM
APEKS-cUTI[77]	Complicated urinary tract infection	300	148	2g q8h with 1-h infusion	Imipenem /cilastatin (1g q8h with 1-h infusion)	152	79	48	25	0	1	18	5	13	3	21	6
APEKS-NP[78]	Nosocomial pneumonia	145	147	2g q8h with 1-h infusion	Meropenem (2g q8h with 3-h infusion)	19	22	48	44	23	24	24	24	7	8	38	42
CREDIBLE-CR[79]	Patients with various infections caused by carbapenem-resistant pathogens	101	49	2g q8h with 1-h infusion	Best available therapy (mainly colistin-based)	2	1	27	12	39	17	12	10	2	0	7	0

RCT, Randomized controlled trial; CFDC, cefiderocol; COM, comparator.

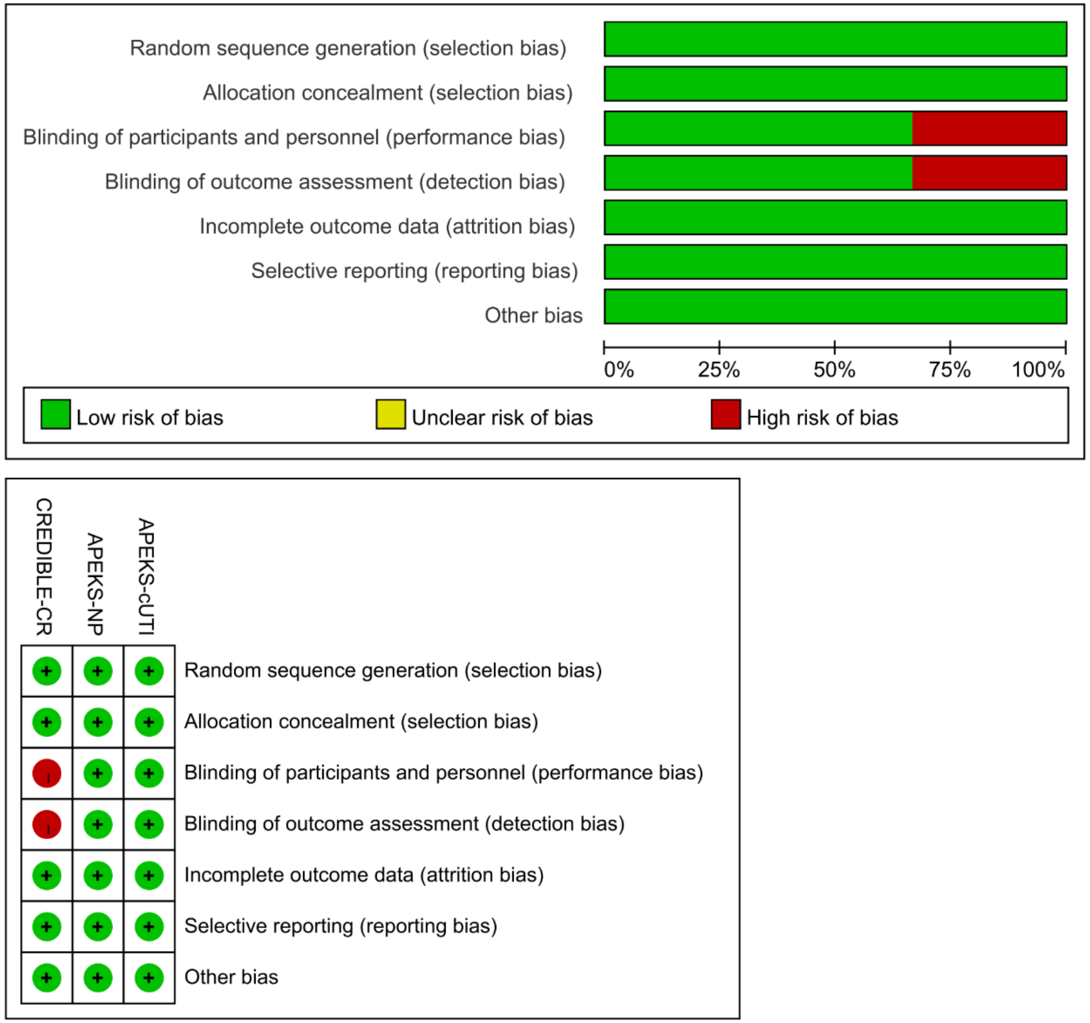

Fig. S1. The risk of bias of three Randomized controlled trials

Table S3. Characteristics of the 78 patients with recalcitrant infections caused by MDR Gram-negative bacteria received salvage treatment or compassionate use of cefiderocol

Reference	Country	Published year	Age	Sex	Main disease status associated with infections	MDR Pathogens	CFDC MICs/inhibition zone diameters	Type of infections	Initial treatments	Reasons for Change	CFDC dosage	CFDC monotherapy/combination therapy	CRRT/HD	clinical outcome	Death	Microbiological eradication	Adverse events
Edgeworth J, et al[81]	UK	2018	78	F	Hydronephrosis secondary to ureteric hematoma	*P. aeruginosa* (XDR)	NA	BSI	COL+GEN+MEM	Treatment failure	2g q8h	COL+MEM	No	success	No	Yes	Leukopenia
Alamarat Z, et al.[82]	USA	2019	15	M	Bone fracture	*P. aeruginosa* (NDM), *K. pneumoniae* (ESBLs)	4	Wound infection	Polymyxin B+TGC/CAZ-AVI+ATM	Neurotoxicity	2g q8h	Monotherapy	No	success	No	Yes	Leukopenia
Stevens R, et al.[83]	USA	2019	46	M	Toe amputation	*P. aeruginosa* (MDR)	0.125	Intra-abdominal infection	COL	Colistin neurotoxicity, treatment failure	750 mg q12h	Monotherapy	Yes	success	No	Yes	No
Trecarichi E, et al.[84]	Italy	2019	Adult	M	H1N1 pneumonia	*A. baumannii* (XDR), *K. pneumoniae* (KPC)	23mm	VAP, BSI	COL+FOS/TGC/COL+SAM/CAZ-AVI+RIF	Colistin toxicity, treatment failure	NA	Monotherapy	No	success	No	Yes	No
Contreras D, et al.[85]	USA	2020	68	F	Renal transplantation	*K. pneumoniae* (OXA-232 and NDM)	NA	Intra-abdominal infection	COL+TGC+CAZ-AVI	Treatment failure	1.5g q12h	Polymixin B+ CAZ-AVI	No	success	Yes, other infection	Yes	No
Dagher	US	2020	57	M	Bone fracture	*A. baumannii*	23mm	Osteomyelitis	COL+MIN/T	Colistin renal	2g q8h	Monother	No	success	No	Yes	No

M, et al.[86]	A					(Carbapenem-resistant)			GC	toxicity, treatment failure		apy					
Falcone M, et al.[87]	Italy	2020	76	F	Burn	*A. baumannii* (Carbapenem-resistant)	0.25	BSI	COL+TGC	All patients experienced treatment failure, 3 developed acute kidney injury	2g q8h	Monotherapy	Yes	Failure	Yes, infection	No	No
			82	M	Burn	*A. baumannii* (Carbapenem-resistant)	0.5	BSI	COL+TGC+FOS		2g q8h	Monotherapy	No	Success	No	Yes	No
			65	F	Burn	*A. baumannii* (Carbapenem-resistant)	0.5	BSI	COL		2g q8h	Monotherapy	No	Failure	Yes, infection	No	No
			33	F	Burn	*A. baumannii* (Carbapenem-resistant)	0.5	BSI	COL+TGC		2g q6h	Monotherapy	No	Success	No	Yes	No
			82	F	Colonic perforation with hemicolectomy	*A. baumannii* (Carbapenem-resistant)	0.25	BSI	COL+TGC+MEM		1.5g q8h	Monotherapy	No	Success	No	Yes	No
			75	F	COVID-19	*A. baumannii* (Carbapenem-resistant)	0.5	BSI	TGC+SAM		2g q6h	Monotherapy	No	Success	No	Yes	No
			79	F	COVID-19	*K. pneumoniae* (NDM), S. maltophilia	1/0.5	VAP	CAZ-AVI+ATM+FOS		2g q6h	Monotherapy	No	Success	No	Yes	No
			44	M	COVID-19	*K. pneumoniae*	1	VAP	COL+FOS		2g q6h	Monotherapy	No	Success	No	Yes	No

						(NDM)											
			77	M	COVID-19	*A. baumannii* (Carbapenem-resistant), *K. pneumoniae* (NDM)	0.12/2	VAP	COL+CAZ-AVI+ATM		1.5g q8h	FOS	Yes	Failure	Yes, infection	Yes	No
			72	M	COVID-19	*A. baumannii* (Carbapenem-resistant)	0.5	VAP	COL+TGC		2g q6h	Monotherapy	No	Success	No	Yes	No
Kufel W, et al.[88]	USA	2020	45	F	Esophageal-pleural fistula	*P. aeruginosa* (XDR)	24mm	Pyothorax	Polymyxin B+CAZ-AVI	Treatment failure	2g q8h	Monotherapy	No	success	No	Yes	No
Lampejo T, et al.[89]	UK	2020	84	M	Wet gangrenous foot.	*K. pneumoniae* (OXA-232 and NDM-5)	NA	Skin and soft tissue infection	COL+TGC	NA	1g q8h	COL	No	success	Yes, Renal failure	Yes	No
Martinez A, et al.[90]	USA	2020	70	F	Cystic fibrosis	*Achromobacter* species	NA	LRTI	COL+TGC	Treatment failure	NA	Monotherapy	No	success	No	NA	No
Oliva A, et al.[91]	Italy	2020	60	M	Haemorrhagic cardiac tamponade	*A. baumannii* (XDR), *K. pneumoniae* (Carbapenem-resistant)	NA	VAP	TMP/SMX	TMP/SMX induced myelotoxicity	1.5g q12h	Monotherapy	Yes	success	Yes, heart failure	Yes	No
			70	F	Diabetes	*A. baumannii* (XDR), *P.*	NA	Pyelonephritis, BSI/sepsis	COL	Treatment failure	2g q8h	Monotherapy	Yes	success	Yes	Yes	No

						aeruginosa (XDR)											
			55	F	severe scoliosis	*A. baumannii* (XDR)	NA	Spinal implant infection	COL+TGC	Colistin renal toxicity; tygecicline induced severe coagulopathy	2g q8h	Monotherapy	No	success	No	Yes	No
Siméon S, et al.[92]	France	2020	67	M	Knee replacement surgery	*Enterobacter hormaechei* (OXA-1)	NA	Joint Infection	COL+TGC	Colistin renal toxicity	2g q8h	Monotherapy	No	success	No	Yes	No
Warner N, et al.[93]	USA	2020	28	NA	Cystic fibrosis	*Achromobacter xylosoxidans*	0.12	LRTI, BSI	NA	NA	2g q8h	TZP	No	Failure	No	No	No
			17	NA	Cystic fibrosis	*Achromobacter xylosoxidans*	1	Peri–lung transplant antibacterial regimen	NA	NA	2g q8h	MEM+TMP/SMX	No	success	No	No	No
			29	NA	Cystic fibrosis	*Achromobacter xylosoxidans*	> 64	Peri–lung transplant antibacterial regimen	NA	NA	2g q8h	CAZ-AVI+TMP/SMX	No	Failure	No	No	No
			41	NA	Cystic fibrosis	*Achromobacter xylosoxidans*	0.06	LRTI, BSI	NA	NA	2g q8h	Eravacycline+Delafloxacin	No	Failure	No	No	No
			25	NA	Cystic fibrosis	*Achromobacter xylosoxidans*	0.06	LRTI	NA	NA	2g q8h	Eravacycline+IMI	No	Failure	No	No	No
			10	N	Cystic fibrosis	*Achromobacte*	32	LRTI	NA	NA	60	MEM-	No	success	No	No	No

				A		*r species*					mg/kg q8h	vaborbactam+Bacteriophage therapy					
			56	NA	Cystic fibrosis	*Achromobacter xylosoxidans*	1	LRTI, Empyema	NA	NA	2g q8h	COL	No	success	No	No	No
			28	NA	Cystic fibrosis	*Achromobacter xylosoxidans*	20mm	LRTI	NA	NA	2g q8h	TMP/SMX+Inhaled AMK	No	Failure	No	No	No
Zingg S, et al.[94]	Switzerland	2020	29	M	Bone fracture	*A. baumannii* (OXA-23), *P. aeruginosa* (VIM), *E. cloacae* (KPC)	NA	Wound infection, Osteomyelitis	NA	NA	NA	CAZ-AVI+ COL	No	success	No	Yes	No
			64	M	Polytrauma	*A. baumannii* (NDM and OXA)	NA	Implant associated infection	NA	NA	2g q8h	COL	No	success	No	Yes	No
			62	M	Thoracic trauma	*A. baumannii* (XDR)	NA	Pleural empyema, osteomyelitis, UTI	NA	NA	2g q8h	COL	No	success	No	Yes	No
Bavaro D, et al.[95]	Italy	2021	64	M	Traumatic right parietal subdural haematoma	*P. aeruginosa* (XDR)	0.5	Wound infection	COL+FOS	Treatment failure	2g q8h	FOS	No	success	No	Yes	No
Bavaro	Italy	2021	68	M	COVID-19	*A. baumannii*	NA	BSI/Sepsis	COL+TGC+	Treatment failure	2g q8h	FOS+TG	No	success	Yes,	Yes	No

D, et al.[96]						(Carbapenem-resistant)			FOS			C			respiratory failure		
			62	F	COVID-19	*A. baumannii* (Carbapenem-resistant)	NA	BSI/Sepsis	MEM+COL	Colistin resistance	2g q8h	COL+MEM	No	success	No	Yes	No
			69	M	COVID-19	*A. baumannii* (Carbapenem-resistant)	NA	BSI/Sepsis	MEM+COL	Treatment failure	2g q8h	COL	No	success	No	Yes	No
			78	M	COVID-19	*A. baumannii* (Carbapenem-resistant)	NA	BSI/Sepsis	MEM+COL+TGC	Treatment failure	2g q8h	TGC	No	success	No	Yes	No
			75	F	COVID-19	*A. baumannii* (Carbapenem-resistant)	NA	BSI/Sepsis	MEM+ COL+ FOS	Colistin toxicity	1.5g q8h	FOS	No	success	No	Yes	No
			38	M	Cerebral hemorrhage	*A. baumannii* (Carbapenem-resistant)	NA	VAP	COL+ FOS+ TGC	Treatment failure	2g q8h	FOS+TGC	No	success	No	Yes	No
			70	M	PTCA due to myocardial Infarction	*A. baumannii* (Carbapenem-resistant)	NA	BSI	MEM+COL+FOS+SAM	Treatment failure	2g q8h	COL+FOS	No	success	No	Yes	No
			64	M	Traumatic subarachnoid hematoma	*K. pneumoniae* (KPC)	NA	Neurosurgical wound infection	COL+FOS	Treatment failure	2g q8h	FOS	No	success	No	Yes	No
			25	M	Subocclusion and volvulus treated	*A. baumannii* (Carbapenem-	NA	Perihepatic Abscess,	MEM+ TGC+FOS	Treatment failure	2g q8h	TGC+FOS	No	success	No	Yes	No

					with gut surgical resection	resistant), E. cloacae complex (MDR)		sepsis									
			60	M	Hepatic transplantation	*K. pneumoniae* (KPC)	NA	Hepatic abscess, BSI/sepsis	TGC+CAZ-AVI+COL	Treatment failure	2g q8h	TGC+COL+ FOS	No	success	No	Yes	No
			43	M	Heart transplantation and MODS	*A. baumannii* (Carbapenem-resistant)	NA	VAP, BSI	COL+ MEM+TGC	Treatment failure	2g q8h	TGC+COL+ FOS	Yes	success	Yes, other infection	Yes	No
			57	M	COVID-19	*A. baumannii* (Carbapenem-resistant)	NA	BSI	MEM+COL	Treatment failure	2g q8h	COL	No	success	Yes, respiratory failure	Yes	No
			68	M	Acute myeloid leukemia	*P. aeruginosa* (XDR)	NA	LRTI	COL+ MEM+ FOS	Treatment failure	2g q8h	FOS	No	success	No	Yes	No
Bleibtreu A et al.[97]	France	2021	54	NA	NA	*P. aeruginosa* (XDR)	2	LRTI	NA	NA	2g q8h (n=9), 750 mg q12h (n=1), 750mg q8h (n=2)	Monotherapy	No	success	No	Yes	Renal failure (n=1), thrombocytopenia (n=1)
			37	NA	NA	*A. baumannii* (XDR)	1	Vascular infection	NA	NA		Monotherapy	No	success	No	Yes	
			54	NA	NA	*A. baumannii* (XDR)	0.5	LRTI, intra-abdominal infection, vascular infection	NA	NA		COL+TGC	No	success	No	Yes	

			72	NA	NA	*P. aeruginosa* (XDR)	4	LRTI	NA	NA		Monotherapy	No	success	No	Yes	
			70	NA	NA	*P. aeruginosa* (XDR)	2	LRTI	NA	NA		Monotherapy	No	success	No	Yes	
			67	NA	NA	*Enterobacter hormaechei* (XDR)	1	LRTI	NA	NA		Monotherapy	No	success	No	Yes	
			31	NA	NA	*K. pneumoniae* (XDR), *P. aeruginosa* (XDR)	0.5	LRTI	NA	NA		COL	No	Failure	No	No	
			49	NA	NA	*P. aeruginosa* (XDR)	8	LRTI, intra-abdominal infection	NA	NA		COL	No	Failure	Yes, infection	No	
			63	NA	NA	*P. aeruginosa* (XDR)	16	LRTI	NA	NA		COL+DOX	No	Failure	Yes, infection	No	
			35	NA	NA	*P. aeruginosa* (XDR)	16	LRTI, UTI	NA	NA		COL	No	Failure	No	No	
			21	NA	NA	*P. aeruginosa* (XDR)	> 32	Bone and joint infection, skin and soft tissue infection	NA	NA		COL	No	Failure	No	No	
			59	NA	NA	*P. aeruginosa* (XDR)	16	LRTI	NA	NA		COL	No	Failure	No	No	

Bodro M et al.[98]	Spain	2021	66	M	Left ventricular assist device implantation	*Achromobacter xylosoxidans*	21mm	Left ventricular assist device infection, BSI	TZP+COL+TGC	Colistin renal toxicity, treatment failure	2g q8h	TZP+TGC	No	success	No	Yes	Thrombocytopenia
			55	M	Bilioportal fistula	*P. aeruginosa* (XDR)	23mm	Biliary tract infection, BSI	COL+MEM	Treatment failure	2g q8h	COL	No	success	No	Yes	No
Borghesi L et al.[99]	Italy	2021	64	M	Malignant mesothelioma	*P. aeruginosa* (Carbapenem-resistant)	0.5	Pleural empyema	GEN+ceftolozane/tazobactam	Treatment failure	2g q8h	COL+FOS	No	success	No	Yes	No
Carney B et al.[100]	USA	2021	33	M	Blast injury	*E.coli* (NDM)	2	Osteomyelitis	COL+TGC	Colistin renal toxicity	2g q8h	TGC	No	success	No	Yes	No
Cipko K et al.[101]	Australia	2021	81	F	Laminectomy	*A. baumannii* (OXA-23)	0.5	Wound infection	COL+TGC+TZP	colistin neurotoxicity	1.5g q8h	Monotherapy	No	success	No	Yes	Acute interstitial nephritis
Chavda A et al.[102]	UK	2021	59	M	Bone fracture	*P. aeruginosa* (IMP)	NA	Osteomyelitis	COL	Colistin renal toxicity	1.5g q8h	Monotherapy	No	success	No	Yes	No
Fratoni A et al.[103]	USA	2021	58	M	Cardiogenic shock	*S. maltophilia*, *E. coli* (ESBLs)	NA	BSI, LRTI	NA	NA	2g q12h	TGC	Yes	success	Yes, organ failure	Yes	No
Grasa CD et al.[104]	Spain	2021	2	F	Burkitt lymphoma	P. aeruginosa (VIM)	22mm	Ecthyma	COL+MEM	Treatment failure	60 mg/kg q8h	Monotherapy	No	success	No	Yes	No
Grande-	Belg	2021	63	M	Diabetic foot-	*P. aeruginosa*	NA	Pancreatic	COL+MEM	Treatment failure	2g q8h	Monother	No	Failure	Yes,	No	No

Perez C et al.[105]	ium				related ulcer	(XDR)		abscess, LRTI				apy			infection		
Klein S et al.[106]	Germany	2021	58	M	liver retransplantation	*K. pneumoniae* (NDM) and Enterobacter cloacae (OXA-48)	NA	BSI, hepatic abscesses	COL+MEM	Colistin neurotoxicity, resistance development	NA	Monotherapy	No	Failure	Yes, infection	No	No
König C et al. [53]	Germany	2021	41	M	Chronic respiratory failure	*P. aeruginosa* (MDR)	0.5	LRTI	NA	NA	1g q8h	NA	Yes	Success	No	Yes	No
			69	F	Esophagectomy	*P. aeruginosa* (MDR)	0.25	LRTI	NA	NA	1g q8h	NA	No	Failure	No	Yes	No
			76	M	COVID-19	*A. baumannii* (MDR)	0.25	LRTI, sepsis	NA	NA	2g q8h	NA	No	Failure	Yes, respiratory failure	Yes	No
			53	M	Autologous Stem Cell Transplantation	*P. aeruginosa* (VIM)	0.125	BSI/Sepsis	NA	NA	2g q8h	NA	Yes	Failure	Yes, septic shock	Yes	No
			50	M	COVID-19 with ECMO	*A. baumannii* (Carbapenem-resistant)	0.25	LRTI	NA	NA	2g q8h	NA	Yes	Failure	Yes, respiratory failure	No	No
Mabayo	UK	2021	66	F	Bone fracture	*A. baumannii*	4	Wound	COL+TGC	Colistin renal	750	TGC	No	success	No	Yes	No

je D, et al.[107]						(OXA-23 and NDM-1)		infection		toxicity	mg q12h						
Mc Gann P, et al.[108]	USA	2021	65	NA	COVID-19 with ECMO	*Providencia rettgeri* (PER-1 and NDM-1)	> 32	BSI	NA	NA	NA	Monotherapy	No	Failure	Yes, multi-organ failure	No	No
Zaidan N, et al.[109]	USA	2021	55	F	COVID-19	*A. baumannii* (XDR)	0.5	LRTI	COL+MEM+SAM+eravacycline	Treatment failure	2g q8h	Sulbactam-durlobactam	No	success	No	Yes	No

NA, not applicable; CFDC, cefiderocol; CRRT, continuous renal replacement therapy; HD, hemodialysis; ECMO, extracorporeal membrane oxygenation; PTCA, percutaneous transluminal coronary angioplasty; MODS, multiple organ dysfunction syndrome; F, female; M, male; MDR, multi-drug resistant; XDR, extensively drug resistant; BSI, bloodstream infection; VAP, ventilator associated pneumonia; LRTI, lower respiratory tract infections; UTI, urinary tract infection; COL, colistin; GEN, gentamicin; AMK, amikacin; MEM, meropenem; IMI, imipenem; TGC, tigecycline; CAZ-AVI, ceftazidime-avibatam; ATM, aztreonam; FOS, fosfomycin; SAM, ampicillin-sulbactam; RIF, rifampin; MIN, minocycline; TMP/SMX, trimethoprim-sulfamethoxazole; DOX, doxycycline; TZP, piperacillin-tazobactam.
